# Automated Single-Sensor 3D Scanning and Modular Benchmark Objects for Human-Scale 3D Reconstruction

**DOI:** 10.3390/s26041331

**Published:** 2026-02-19

**Authors:** Kartik Choudhary, Mats Isaksson, Gavin W. Lambert, Tony Dicker

**Affiliations:** 1 Department of Mechanical Engineering and Product Design Engineering, Swinburne University of Technology, Hawthorn, VIC 3122, Australia; 2 Iverson Health Innovation Research Institute, Swinburne University of Technology, Hawthorn, VIC 3122, Australia; 3 Faculty of Health, Medicine and Behavioural Sciences, The University of Queensland, Brisbane, QLD 4006, Australia

**Keywords:** 3D scanning, single-sensor system, human body reconstruction, automation, point cloud registration, geometric benchmarking

## Abstract

High-fidelity 3D reconstruction of human-sized objects typically requires multi-sensor scanning systems that are expensive, complex, and rely on proprietary hardware configurations. Existing low-cost approaches often rely on handheld scanning, which is inherently unstructured and operator-dependent, leading to inconsistent coverage and variable reconstruction quality. This limitation necessitates the need for a controlled, repeatable, and affordable scanning method that can generate high-quality data without requiring multi-sensor hardware or external tracking markers. This study presents a marker-less scanning platform designed for human-scale reconstruction. The system consists of a single structured-light sensor mounted on a vertical linear actuator, synchronised with a motorised turntable that rotates the subject. This constrained kinematic setup ensures a repeatable cylindrical acquisition trajectory. To address the geometric ambiguity often found in vertical translational symmetry (i.e., where distinct elevation steps appear identical), the system employs a sensor-assisted initialisation strategy, where feedback from the rotary encoder and linear drive serves as constraints for the registration pipeline. The captured frames are reconstructed into a complete model through a two-step Iterative Closest Point (ICP) procedure that eliminates the vertical drift and model collapse (often referred to as “telescoping”) common in unconstrained scanning. To evaluate system performance, a modular anthropometric benchmark object representing a human-sized target (1.6 m) was scanned. The reconstructed model was assessed in terms of surface coverage and volumetric fidelity relative to a CAD reference. The results demonstrate high sampling stability, achieving a mean surface density of $0.760\;{\mathrm{points}/\mathrm{mm}}^{2}$ on front-facing surfaces. Geometric deviation analysis revealed a mean signed error of −1.54 mm (σ= 2.27 mm), corresponding to a relative volumetric error of approximately 0.096% over the full vertical span. These findings confirm that a single-sensor system, when guided by precise kinematics, can mitigate the non-linear bending and drift artefacts of handheld acquisition, providing an accessible yet rigorously accurate alternative to industrial multi-sensor systems.

## 1. Introduction

Accurate three-dimensional (3D) representation of human subjects is a growing requirement across domains such as ergonomics, virtual prototyping, digital health, advanced manufacturing, and human–robot interaction [1,2]. The ability to digitise human geometry—whether of a live subject or an anthropometric surrogate—with high fidelity and reproducibility offers significant value for design validation, simulation-based analysis, and physical interaction modelling. However, acquiring high-quality 3D data at human scale poses significant challenges due to large scanning volume, potential for occlusions, surface complexity, and the inherent difficulty of frame-to-frame alignment in multi-view capture scenarios [3,4].

Traditional approaches to 3D scanning at human-scale typically fall into two categories: handheld scanning and automated multi-sensor systems. Handheld scanners are widely adopted due to their accessibility, ease of use in applications requiring mobility and low cost. However, their performance is limited by unstructured sensor trajectories, uneven frame overlap, and a lack of controlled pose acquisition. Munkelt et al. [5] reported that the absence of structured guidance during handheld acquisition can cause significant deviations compared to scans from stationary systems. Moreover, Yang et al. [6] demonstrated that insufficient frame-to-frame overlap frequently results in registration errors and requires additional post-processing to achieve usable reconstructions. Without controlled poses, handheld systems also depend heavily on the operator’s ability to maintain stable orientation and distance from the subject, which introduces variability in scan quality. Anderson et al. [7] showed that unregulated scanner placement significantly compromises reconstruction completeness, particularly in applications requiring high fidelity.

To improve consistency and reduce the operator dependence associated with handheld scanning, automated multi-sensor systems have been developed. These high-end industrial solutions typically employ arrays of synchronised structured light or laser sensors to capture the subject instantaneously from multiple angles. While highly accurate, they are constrained by significant limitations. First, the high cost of multi-sensor hardware and proprietary software restricts their accessibility. For example, Geng [8] notes that such systems require specialised components and substantial computational resources, resulting in considerable financial investment that can be prohibitive for smaller institutions. Second, their physical size and setup complexity limit portability. Multi-sensor photogrammetry booths, while effective, require large synchronised camera arrays to ensure simultaneous capture and rigorous calibration routines, rendering them unsuitable for compact or low-cost deployments. Beeler et al. [9] demonstrated a high-fidelity facial scanning system but noted the need for a carefully controlled multi-camera environment, illustrating the strict lighting and spatial constraints that limit such systems to laboratory settings. Finally, these systems often lack flexibility. Zollhöfer et al. [10] observed that many automated arrays struggle with texture-less surfaces and require carefully calibrated environments, making them impractical for dynamic applications. In contrast to these industrial solutions, simple single-sensor automated turntables exist for small-scale objects. However, these desktop systems lack the vertical range of motion necessary to cover a human-sized volume [11]. Consequently, capturing a standing subject typically requires choosing between a complex multi-sensor array or a manual operator to move the sensor vertically, which reintroduces the stability issues of handheld scanning.

Beyond hardware limitations, a critical challenge common to both handheld and automated 3D scanning systems is the alignment of multiple views in the absence of distinctive visual features. In such contexts, geometry-based registration methods are commonly employed, relying on spatial correspondences rather than colour cues. However, the performance of these algorithms depends heavily on the uniqueness of the underlying geometry. As Rusinkiewicz and Levoy [12] and Zhu et al. [13] noted, registration becomes unstable when the object exhibits vertical translational symmetry—such as the smooth, cylindrical shape of a human limb. In these “feature-poor” scenarios, standard algorithms cannot reliably distinguish between adjacent vertical sections, causing the point cloud to slide along the axis of symmetry. This registration failure, often referred to as “telescoping” or longitudinal drift, results in severe geometric contraction and model collapse [14]. To mitigate this, traditional workflows often rely on applying adhesive photogrammetry markers to the subject to create artificial tracking points. However, this process is intrusive and necessitates physical contact with the subject, limiting its viability in medical or ergonomic assessments where a completely non-invasive acquisition workflow is preferred [15].

Compounding these hardware and algorithmic challenges is the scarcity of standardised physical benchmarking objects. Most existing validation efforts rely on synthetic datasets, small-scale calibration targets, or real-world objects with uncontrolled surface properties and dimensions [16,17]. While these approaches are useful for assessing algorithmic robustness in isolated cases, they are insufficient for controlled benchmarking of scanning systems operating at human scale [18]. The absence of scalable, reconfigurable, and anthropomorphically relevant physical test objects has limited the reproducibility of experimental evaluations and constrained the development of scanning platforms optimised for large-object reconstruction [19,20].

To mitigate the high costs of multi-sensor systems, the operator dependence of handheld scanning, and the intrusiveness of marker-based tracking, this work presents a marker-less automated scanning platform tailored for human-scale object reconstruction. The system integrates a single structured-light scanner mounted on a vertical linear drive, synchronised with a motorised turntable. This kinematic configuration allows the subject to be systematically captured from controlled elevations and viewing angles, ensuring comprehensive volumetric coverage. By enforcing a structured acquisition path with user-defined overlaps, the system improves the reliability of geometry-based registration methods [21]. Unlike handheld or fixed setups, this design supports repeatable multi-view capture across diverse object configurations without relying on external tracking systems or predefined feature correspondences.

Complementing the 3D scanning platform, a set of modular benchmarking objects was developed using anthropometric principles. These 3D-printed objects consist of reconfigurable cubic elements that can be stacked to represent various body segment heights and configurations, offering control over both geometric complexity and physical scale [20]. The combination of pose-aware data acquisition and structured physical benchmarking provides a reproducible foundation for evaluating 3D reconstruction pipelines in scenarios where texture is absent and surface features are minimal [18].

The main contributions of this study are as follows:

1. A controlled single-sensor scanning platform is developed, combining a vertical linear drive to translate the sensor with a motorised turntable to rotate the scanned object, thereby generating a repeatable, high-density acquisition path covering the full volume of the subject.
2. A modular anthropometric benchmarking object is introduced, consisting of 3D-printed reconfigurable cubic units designed to replicate human-sized dimensions and provide a physically consistent target for system-level evaluation.
3. A sensor-assisted registration framework is implemented, which utilises kinematic feedback from the motion stages to initialise and constrain the alignment of the partial scans of the object. This approach eliminates vertical drift in feature-poor data without requiring external tracking markers.
4. A quantitative geometric correspondence analysis is presented, comparing the reconstructed model to a CAD reference to characterise volumetric fidelity and surface coverage relative to the acquisition trajectory of the 3D scanner.

The remainder of this paper is organised as follows. Section 2 outlines the methodology, covering the hardware design, software architecture, and reconstruction workflow of the proposed scanning system. Section 3 presents the evaluation procedure and results. Section 4 concludes the paper and outlines directions for future work.

## 2. Methods

### 2.1. System Hardware

**Figure 1:**
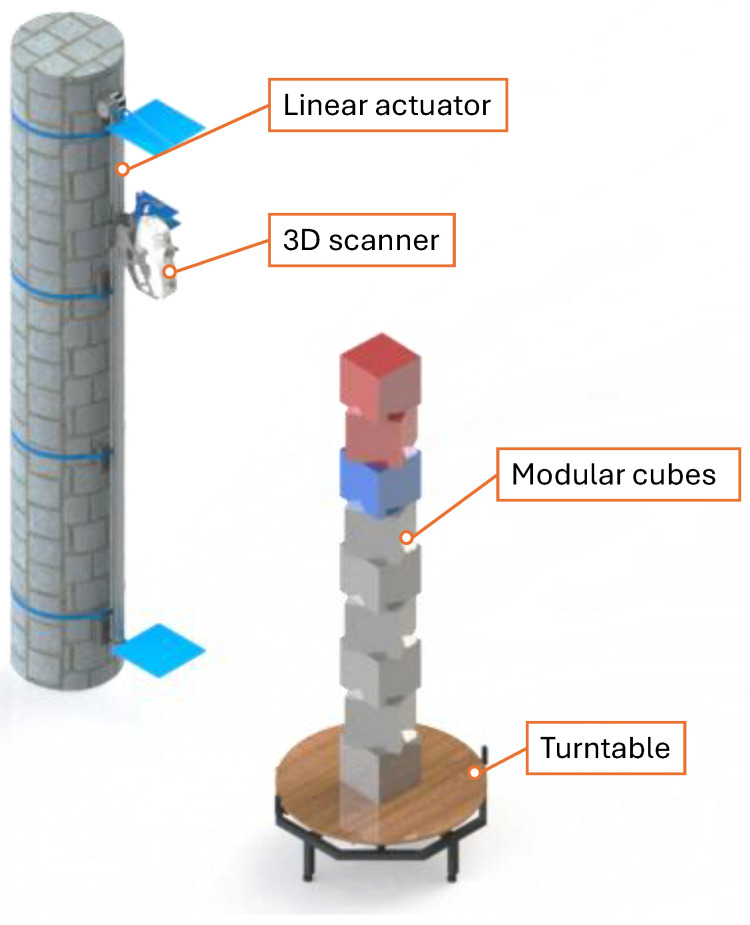
The developed 3D scanning system for capturing surface data of human-sized objects, showing the 3D scanner mounted on a linear actuator, a motorised turntable, and modular cubes.

Figure 1 illustrates the key components of the 3D scanning system, which include a 3D scanner, a motorised linear actuator, a motorised turntable, and modular cubes.

#### 2.1.1. 3D Scanner and Scanner Mount

**Figure 2:**
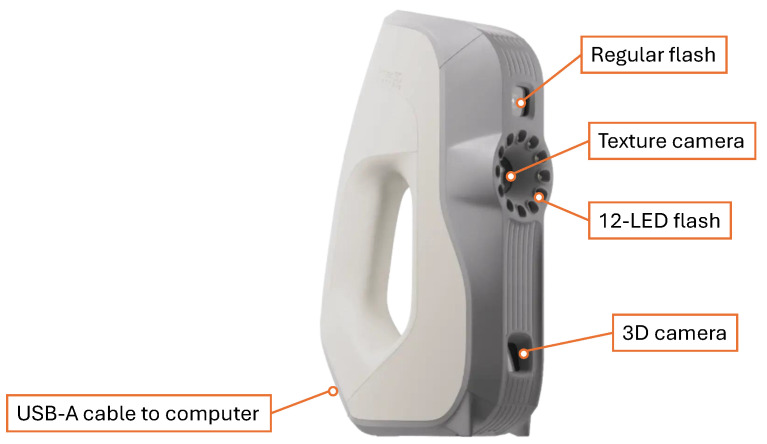
Components of the Artec Eva Lite Scanner. The diagram highlights key components: a regular flash, a textured camera (hardware present, inactive in the Eva Lite model), a 12-LED flash, a 3D camera, and a USB cable to the computer.

The system uses Artec Eva Lite, shown in Figure 2, a portable scanner using structured light. This scanner is well-known for its applications in several benchmarking studies of scanned objects [22,23]. It has a 3D point accuracy of 0.1 mm and a resolution of up to 0.5 mm. It operates well within a range of 0.4 m to 1 m and can capture volumetric contents up to 61,000 cm^3^, making it well-suited for scanning human figures and objects of comparable size.

**Figure 3:**
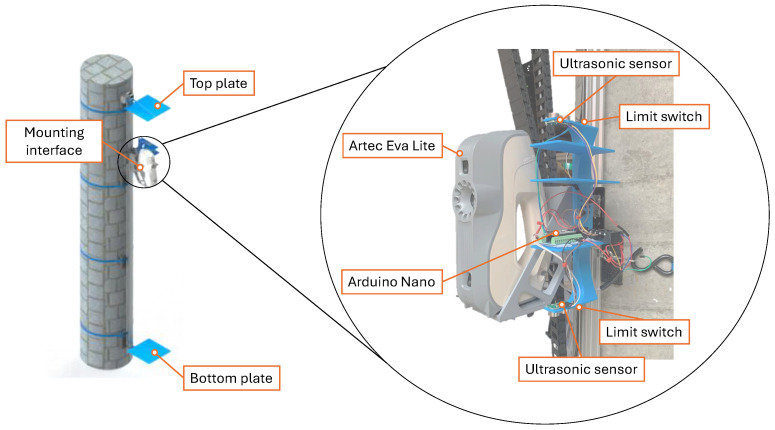
Mounted Eva Lite scanner with key components labelled, including limit switches, ultrasonic sensors, Arduino Nano microcontroller, and scanner mount.

A custom mounting interface was designed to rigidly couple the Artec Eva Lite scanner to the linear actuator’s carriage. Given Eva Lite’s angular field of view of 30° in height and 21° in width, which captures a broader vertical range, it is mounted in a vertical orientation, as shown in Figure 2. The mount was manufactured using 3D-printed PLA+ with 100% infill, ensuring sufficient structural rigidity to minimise vibration artefacts during acquisition. As shown in Figure 3, the assembly incorporates two mechanical limit switches positioned at the vertical extremities of the mounting interface. These switches serve a dual purpose: they indicate when the scanner has reached the physical limits of travel and assist in calibrating the linear actuator during the initial run. During calibration, the mount completes a full cycle from top to bottom, allowing the system to measure the total distance covered.

Two ultrasonic distance sensors are positioned facing up and down to measure the distance from the top and bottom. These sensors prevent system overloading or excessive travel beyond the specified limits. If the mount exceeds the designated travel distance or surpasses the threshold error of 1 mm, the system will automatically adjust the travel distance. In cases where the error exceeds 5 mm, the system will initiate a rapid motor lock procedure. This procedure causes the actuator to stop, effectively holding the enclosure assembly in place and stopping further movement.

All communication and control of the sensors in the scanner mount are managed by an Arduino Nano. This microcontroller was chosen for its compact size, 16 MHz clock speed, and capability to serve as both a reader and writer interface using the Inter-Integrated Circuit (I2C) protocol.

#### 2.1.2. Vertical Linear Actuator

**Figure 4:**
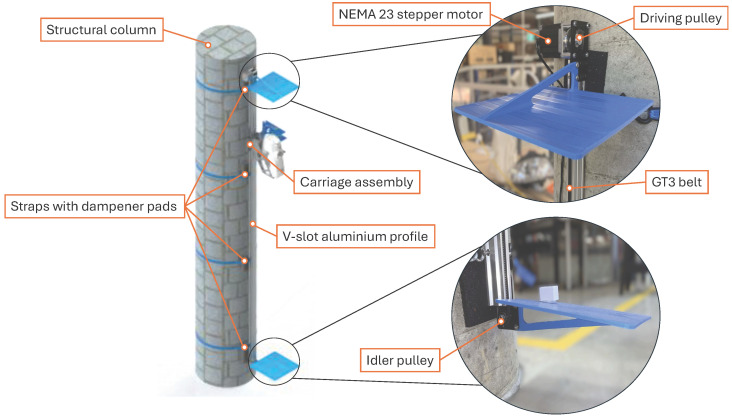
Linear actuator design featuring a NEMA 23 stepper motor, carriage plate on a 20 mm × 40 mm V-slot aluminium profile, GT3 belt, and vibration-dampening straps.

Vertical translation of the carriage is achieved using a custom belt-driven linear motion stage, as illustrated in Figure 4. The system utilises a wheeled carriage plate that traverses a 20 mm × 40 mm aluminium extrusion profile. Motion is transmitted via a GT3 timing belt driven by a NEMA 23 stepper motor, which is coupled to a 20-tooth drive pulley and an opposing idler. The entire assembly is secured to a rigid structural column using dampened mounting straps to minimise vibration transfer. This design provides a usable vertical stroke of 1.7 m, sufficient to scan the full height of a standing subject.

The NEMA 23 motor generates 1.26 Nm of holding torque, capable of driving vertical loads significantly exceeding the 1 kg mass of the sensor assembly. The motor is controlled by an Arduino Nano via a DM556 industrial stepper driver. While the driver supports fine micro-stepping, it was configured for half-stepping mode (400 steps/revolution). This setting was selected to mitigate the mechanical resonance inherent in full-step operation while maintaining higher incremental torque compared to high-resolution micro-stepping modes. The resulting effective linear resolution of 0.15 mm per step provides sufficient positional granularity for the required overlapping acquisition.

#### 2.1.3. Motorised Turntable

**Figure 5:**
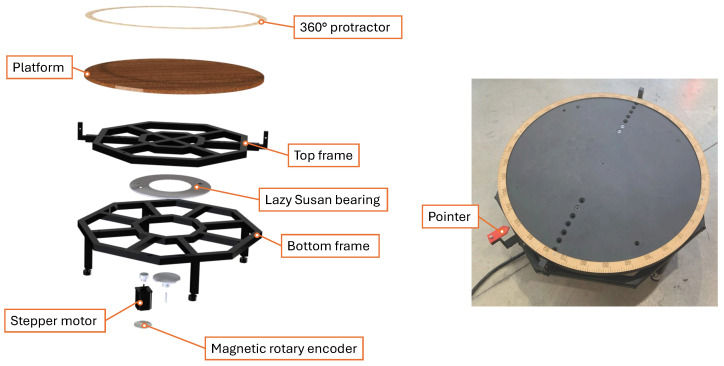
Motorised turntable design for the scanning system, presented with an exploded view (**left**) and an assembled view (**right**). Key components include the top and bottom frames, a lazy Susan bearing, a NEMA 34 stepper motor, a magnetic rotary encoder, a platform, a 360° protractor, and a pointer.

Figure 5 illustrates the turntable designed for this system, highlighting its key components. The turntable incorporates a high-load Lazy Susan bearing with a load capacity of 225 kg. Its structure is reinforced by a frame constructed from a 20 mm square hollow section (SHS), ensuring the custom-build turntable’s stability and rigidity. The frame is divided into two parts: the bottom frame, which is stationary and secured to the bottom of the bearing, and the top frame, which attaches to the bearing’s upper part and rotates. A 20 mm thick, 650 mm diameter composite timber substrate mounted to the top frame serves as a stable platform for positioning objects. The turntable surface was levelled using a spirit level to ensure planar alignment during scanning.

The turntable is actuated by a NEMA 34 stepper motor, driven by a DM860T digital stepper driver and controlled by a second Arduino Nano. The motor, which provides 12 Nm of torque, is vertically mounted on the lower frame, and its shaft is directly coupled to the upper frame through an adaptor plate to enable efficient transfer of motion. This configuration allows the motor to overcome bearing friction even when supporting loads of up to 120 kg. For smooth and precise rotational control, the stepper driver is configured to deliver 20,000 pulses per revolution, resulting in an effective step angle of 0.018°.

To monitor the rotation angle of the turntable, the system uses an AS5600 magnetic encoder. The encoder comprises a dual-channel Hall effect sensor mounted to the lower section of the motor using a 3D-printed holding plate. With a 12-bit resolution, the sensor provides an angular measurement precision of 0.0879°. Additionally, a laser-cut protractor is mounted around the circumference of the plywood platform, together with a fixed pointer that indicates the motor’s current angle. With a resolution of 1°, this scale offers visual feedback to confirm that the turntable reaches the commanded rotation.

#### 2.1.4. Modular Cubes

The modular cubes used in this study function as anthropometrically relevant evaluation objects. To ensure the physical target represents the spatial scale of a human subject, the cube dimensions were derived directly from biomechanical data. The side length of 177 mm was specifically selected to match the average self-selected stance width (177 ± 47 mm) reported by Schmidle et al. [24]. Consequently, when stacked, the cubes create a reference volume with a footprint and frontal profile that approximate the typical ground area occupied by a standing human.

**Figure 6:**
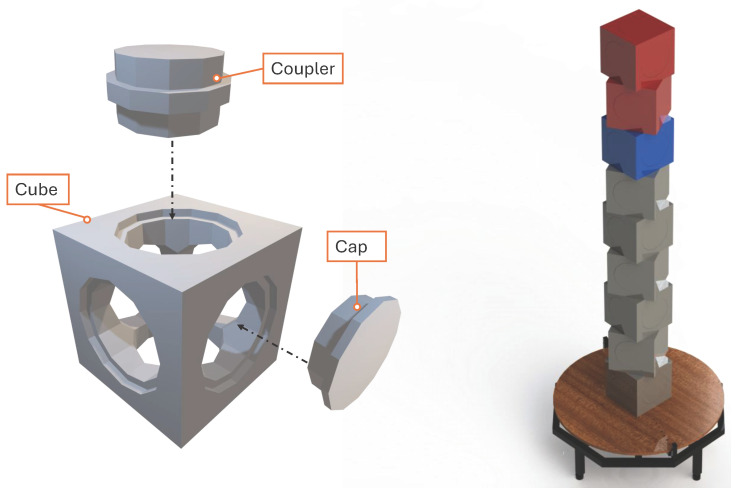
Modular cubes used as the evaluation object in this study. The cube body, coupler, and cap, with a dodecagon interface on all cube faces (**left**) and the stacked configuration with a 30° rotation between layers, used for the scanning experiments in this study (**right**).

Each cube has a dodecagon cut-out on all six faces. This cut-out is the interface used for assembly. It can hold either a coupler or a cap as shown in Figure 6. The coupler links one cube to another and prevents lateral movement during scanning operation. The cap is used to close a face when no additional cube is added. All parts are 3D-printed with fine layer settings so they can be produced and replaced easily.

Figure 6 shows the cube configuration used in this study. Nine cubes were stacked with a 30° rotation between layers providing a simple-to-assemble layout that can be reproduced across trials. Although only this arrangement is used here, the modular design allows other patterns if different geometric conditions or levels of occlusion are needed. The CAD model of the cubes enables direct comparison between the scanned output and the original geometry for evaluation.

**Figure 7:**
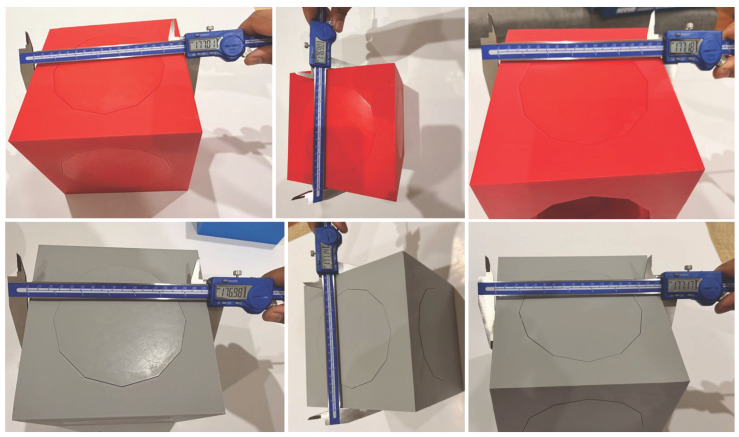
Physical measurement of the modular cubes. Digital caliper measurements of the cubes’ sides. These measurements were used to quantify dimensional deviations of the physical benchmark relative to the nominal CAD geometry.

Although the modular cubes were designed to nominal CAD dimensions, the physical benchmark geometry inevitably incorporates manufacturing and assembly tolerances. To quantify this effect, direct digital caliper measurements were performed on the side lengths of all printed cubes as shown in Figure 7. While the nominal CAD side length is 177.00 mm, the physical measurements ranged from a minimum of 176.82 mm to a maximum of 177.18 mm. The calculated mean side length was 177.03 mm, indicating a negligible mean positive bias of +0.03 mm relative to the nominal CAD geometry.

These deviations arise from a combination of FDM printing tolerances, post-print shrinkage, and assembly-induced offsets. The observed spread of up to ±0.18 mm demonstrates that the CAD benchmark represents an idealised nominal geometry rather than the exact physical envelope of the assembled object. This establishes a non-algorithmic source of local geometric deviation that must be considered when interpreting cloud-to-mesh errors relative to CAD.

### 2.2. System Software

The system software coordinates the movement of the linear actuator and turntable, manages sensor feedback, and controls the timing of each scan. Moreover, it integrates the motion control and the operator interface into a unified command sequence. This ensures that the system automatically halts the scanner at the exact pre-defined locations required for data capture, enforcing a consistent and repeatable scanning path.

#### 2.2.1. Motion Control Architecture

**Figure 8:**
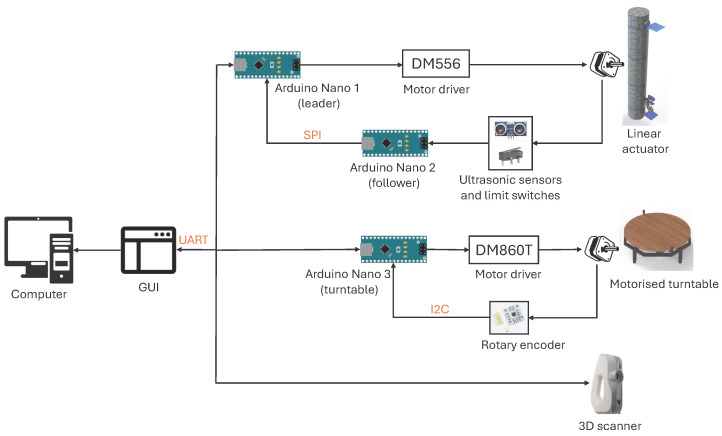
Schematic diagram of the motion control architecture, showing the integration of microcontroller, sensor inputs, motor drivers, and computer communication.

The motion control architecture manages the linear and rotational movement of the scanning platform. The system uses three Arduino Nano microcontrollers, as shown in Figure 8. One Arduino controls the linear actuator, a second controls the turntable, and a third monitors the ultrasonic and limit switch signals, ensuring the distance and boundary information used for vertical motion is available without delay.

Data exchange between the host workstation and microcontrollers is managed via Universal Asynchronous Receiver-Transmitter (UART) interfaces established over USB. The system employs a command–response handshake protocol, where the workstation dispatches motion instructions and waits for execution confirmation flags from the controllers. Locally, the linear actuator’s microcontroller (Leader) receives real-time proximity and limit status from the sensor-monitoring unit (Follower) via a dedicated Serial Peripheral Interface (SPI) bus, ensuring immediate response to safety boundaries. Simultaneously, the turntable controller validates angular displacement by polling the rotary encoder via an Inter-Integrated Circuit (I^2^C) interface. The Eva Lite scanner operates on a separate high-bandwidth USB channel, controlled directly by the Artec Software Development Kit (SDK).

It is worth noting that while this distributed architecture relies on software-level handshakes rather than hardware-level real-time synchronisation, the stop-and-scan acquisition logic renders strict latency bounds unnecessary. Because the scanner and turntable are stationary during the exposure window, the system is insensitive to communication millisecond-level delays. The command–response protocol ensures that no acquisition is triggered until position confirmation is received from all axes, thereby prioritising state consistency over continuous-motion speed.

#### 2.2.2. User Interface

**Figure 9:**
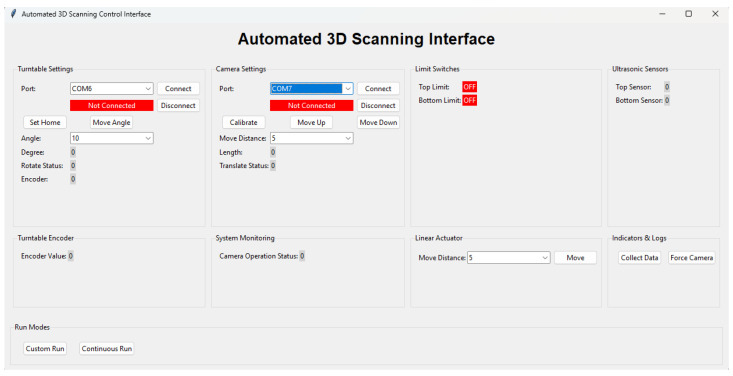
User interface for controlling and monitoring the scanning system. The interface displays motion parameters, sensor readings, and system status in real time.

A graphical user interface (GUI) provides access to the system controls and monitoring. Figure 9 shows the interface layout. The GUI, written in Python v3.13, includes options for connecting to each Arduino, setting movement parameters, and initiating the scan. Real-time displays show encoder values, distance measurements, and the state of the limit switches. Colour-coded indicators identify connection states and error conditions.

Automated scan modes move the system through the full pose sequence with minimal user interaction. Serial communication is handled in a separate thread to maintain interface responsiveness. Safety measures include continuous monitoring of all switches, detection of abnormal distance readings, and the availability of reset controls.

#### 2.2.3. Data Acquisition Workflow

The data collection process commences with a homing routine that translates the scanner to the upper limit of the linear actuator and rotates the turntable to its angular origin. The operator selects the height and rotation increments using the user interface, after which the system moves through the programmed sequence. At each pose, the linear actuator and turntable move to the required position and return a confirmation. Once both are stationary, the Artec SDK captures the frame. Because the scanning trajectory, dwell time, and overlap are governed entirely by the microcontroller firmware, the acquisition process is fully deterministic and independent of the operator. This eliminates human handling as a variable, ensuring that identical scan parameters yield identical sensor poses regardless of the user initiating the sequence.

The scan data is saved using a naming format that encodes the turntable angle and scanner height, enabling efficient organisation and retrieval during reconstruction. Sensor values are monitored and logged throughout the sequence. If a limit switch is triggered or an ultrasonic reading is outside the expected envelope, the linear actuator controller stops the motion, reports the event and corrects it ensuring that the scan remains within safe operating limits.

After the scan is complete, a Python script checks the output directory for empty or incomplete files and flags any issues for review. This ensures that only valid frames are used in the reconstruction process.

All data acquisition trials were conducted in a climate-controlled laboratory environment with an ambient temperature of 23±1 °C. This environmental stability ensures that thermal expansion of the aluminium structural column remains negligible (<0.1 mm) over the duration of a scan sequence.

### 2.3. Registration Pipeline

Each independent mesh is first converted into a uniformly sampled point cloud using a custom Python script. A critical challenge in scanning repetitive vertical structures, such as the modular cube assembly, is geometric ambiguity. Standard registration algorithms, which rely on unique surface features to track position, often fail when consecutive frames exhibit identical geometry.

**Figure 10:**
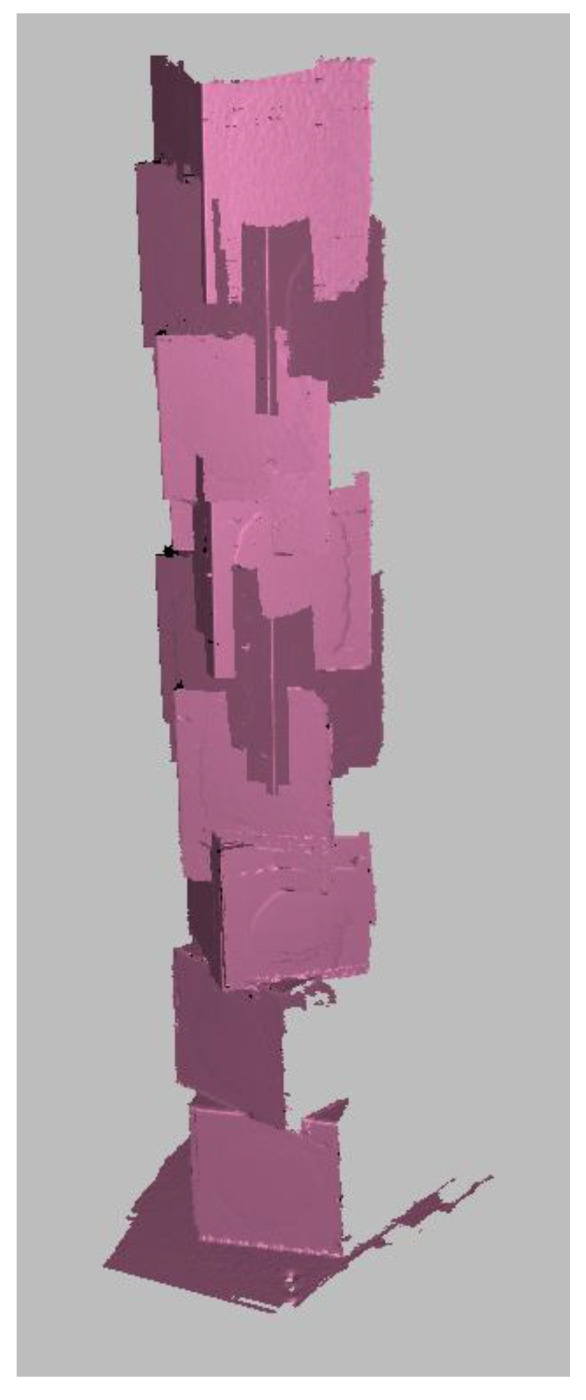
The effect of geometric aliasing on standard registration. Without external kinematic constraints, the repetitive geometry of the stacked cubes causes the commercial feature-tracking algorithm (Artec Studio) to misidentify vertical position, resulting in a “telescoping” collapse of the model height.

**Figure 11:**
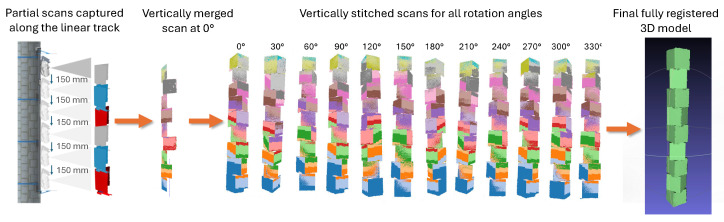
Overview of the reconstruction pipeline. The first stage shows the partial scans captured as the scanner moves along the linear track. These partial scans are merged vertically to form the 0° scan. The process is repeated for all rotation angles, producing a set of vertically stitched scans from 0° to 330°. The final stage shows the complete merged model obtained after rotational ICP registration of all angle-wise scans.

As illustrated in Figure 10, preliminary reconstruction attempts using unconstrained feature tracking resulted in severe vertical drift. The algorithm, unable to distinguish between identical stacked cubes, converged into local minima, causing the 1.6 m object to collapse into a compressed mesh. To resolve this, the proposed pipeline follows a staged alignment strategy that leverages the known kinematics of the scanning platform to constrain the reconstruction as shown in Figure 11.

First, point clouds corresponding to different scanner heights are aligned to form a single vertical segment for each turntable angle. This process utilises a sensor-assisted initialisation strategy: the precise vertical displacement (Δz), determined by the calibrated steps of the linear actuator, provides the initial coarse alignment for each frame. An Iterative Closest Point (ICP) algorithm is then applied to perform fine registration, refining the vertical offset. This ensures that the stitched vertical segment maintains high linearity without the accumulation of angular drift [12,25].

After vertical alignment is completed for all rotation angles, a second registration step integrates these angle-wise point clouds into a single global model. Similarly, the discrete angular position (θ) recorded by the rotary magnetic encoder is used to generate the initial transformation matrix for each scan segment. This provides a precise coarse alignment, effectively placing the point cloud within the convergence basin of the global optimum. Following this initialisation, a constrained ICP is applied to perform fine registration. In this stage, the algorithm solves only for the residual angular misalignment (Δθ) caused by minor mechanical compliance. By coupling hardware telemetry with the software pipeline for both linear and rotational stages, this approach mitigates the risk of the ICP sliding into incorrect local minima [12,26].

Following registration, a weak Laplacian smoothing filter is applied to the assembled model to suppress isolated surface artefacts. This step is tuned to improve surface regularity while minimising the volumetric shrinkage typically associated with smoothing operations on convex geometries [27].

### 2.4. Sampling Parameter Selection and Stability Analysis

The angular and vertical sampling parameters used in this study were selected empirically based on stable ICP convergence and consistently high overlap fitness. To quantify how coarser sampling affects registration stability, additional acquisitions were performed using varying rotation steps (10–40°) and vertical steps (50–200 mm) under otherwise identical conditions.

**Figure 12:**
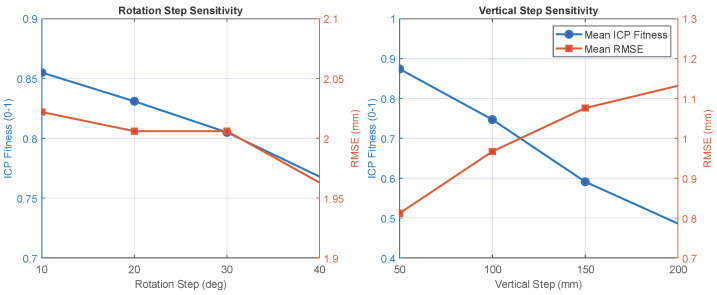
Registration stability analysis. Effect of rotation step size (**left**): Stability degrades monotonically, with a pronounced drop in fitness and repeatability beyond 30°. Effect of vertical step size (**right**): A sharp decline in convergence quality (dropping fitness) is observed beyond 150 mm, identifying it as the stability limit.

For each configuration, the mean ICP fitness and residual RMSE were extracted from the registration logs. The results are visualised in Figure 12.

As shown in Figure 12 (left), fitness decreases monotonically with angular step size, dropping from 0.855 at 10° to 0.768 at 40°. Although mean RMSE remains relatively constant (right axis), analysis of the logs revealed that the standard deviation of this error increases significantly at 40°, reflecting reduced repeatability.

Similarly, Figure 12 (right) demonstrates the impact of vertical sampling. A marked degradation in registration stability is observed between 150 and 200 mm. At 150 mm, the inter-frame overlap remains sufficient to maintain mean fitness above 0.5, whereas at 200 mm the fitness drops below 0.5.

Based on these trade-offs between convergence robustness and acquisition efficiency, the parameters of 30° rotation and 150 mm vertical step were selected as the sparsest stable sampling regime for all subsequent experiments.

### 2.5. Evaluation Metrics

To quantify the performance of the scanning platform, two primary metrics were defined: surface coverage analysis and geometric accuracy assessment. These two metrics were selected to decouple visibility limitations from geometric fidelity. Surface coverage quantifies whether the acquisition trajectory provides sufficient line-of-sight access to all externally visible regions of the target, independent of registration quality. Cloud-to-mesh deviation, in contrast, measures the volumetric accuracy of the reconstructed geometry relative to a known reference. Together, these metrics capture both data completeness and geometric correctness, which are the two dominant failure modes in large-scale structured-light reconstruction [28].

#### 2.5.1. Surface Coverage Assessment

Surface coverage was evaluated by analysing the spatial distribution and local density of points in the final reconstructed model. The merged point cloud was processed in CloudCompare [29] to compute a radius-based density scalar field. For every point $P_{i}$ in the cloud, the surface density ρ is defined as the count of neighbouring points *N* falling within a spherical volume of fixed radius *R*:

(1) $$\rho \left(P_{i}\right)=\sum_{j=1}^{M}I\left(d\left(P_{i},P_{j}\right)<R\right)\;$$

where $d(P_{i},P_{j})$ is the Euclidean distance between point $P_{i}$ and its neighbour $P_{j}$, and I is the indicator function. In this study, a kernel radius of R=6 mm was empirically selected. This value corresponds to approximately 5 to 10 times the typical inter-point spacing of the raw scan data (∼0.5–1.0 mm), ensuring a statistically significant neighbourhood for density estimation while remaining small enough to resolve local occlusion boundaries on human-scale features. The resulting density map serves as a quantitative proxy for visibility, distinguishing between well-observed planar surfaces (high density) and regions affected by occlusion or grazing angle acquisition (low density).

#### 2.5.2. Geometric Accuracy Assessment

Geometric accuracy was assessed using a Cloud-to-Mesh (C2M) signed distance comparison between the reconstructed point cloud and the reference CAD model of the modular cube assembly. Prior to calculation, the raw point cloud was uniformly sub-sampled to ensure an even weight distribution across the surface. The sub-sampled cloud was then aligned to the CAD reference using a rigid ICP algorithm to minimise global transformation errors. Following alignment, the signed Euclidean distance $d_{s}$ was computed from each measured point $P_{m}$ to the nearest triangular face $T_{n}$ on the CAD mesh:

(2) $$d_{s}\left(P_{m}\right)=\min_{T_{n}\in M}∥P_{m}-{\mathrm{proj}}_{T_{n}}\left(P_{m}\right)∥\cdot \mathrm{sgn}\left(n\cdot \left(P_{m}-{\mathrm{proj}}_{T_{n}}\left(P_{m}\right)\right)\right)$$

where *n* is the surface normal vector at the projection point. This metric generates a deviation field where positive values indicate geometry reconstructed outside the reference hull, and negative values indicate geometry inside the reference hull, allowing for the identification of systematic volumetric biases such as smoothing shrinkage or drift.

## 3. Results and Discussion

The results from the experimental evaluation quantifies the performance of the scanning platform.

### 3.1. Surface Coverage Analysis

**Figure 13:**
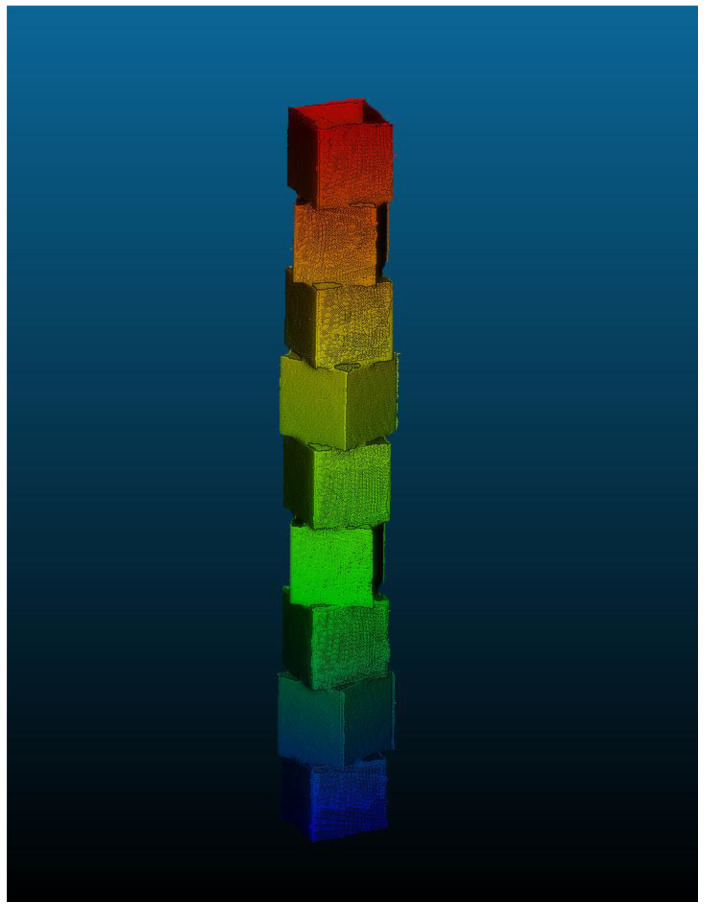
Final registered point cloud of the modular cube assembly used for analysis.

The spatial distribution of the reconstructed point cloud, visualised in Figure 13, reveals distinct visibility patterns driven by the object’s stepped geometry.

**Figure 14:**
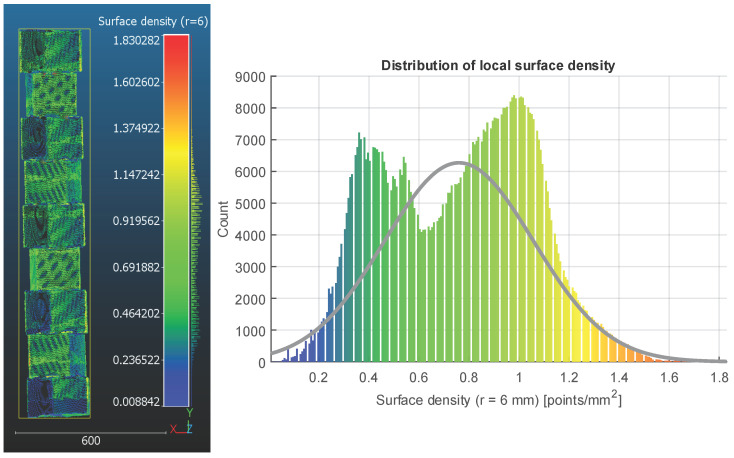
The surface density characteristics of the reconstructed model. The density map computed using a 6 mm neighbourhood radius highlights higher point density on the front-facing planar faces and reduced density along recessed or inward-facing surfaces created by the 30° offsets (**left**). The accompanying histogram illustrates the full density distribution together with a fitted Gaussian curve (mean = 0.760 points/mm^2^, SD = 0.298 points/mm^2^), showing two broad modes that correspond to well-exposed surfaces and partially occluded regions (**right**).

The computed density map (Figure 14) confirms that the scanning trajectory consistently sampled the front-facing planar faces, which appear as high-density regions. Conversely, lower values occur in recessed joints and inward-facing interfaces created by the 30° rotation between layers.

The histogram of surface density (Figure 14, right) exhibits a non-symmetric distribution. A clear concentration of points lies between approximately 0.4 and 1.2 points/mm^2^, corresponding to the well-exposed vertical faces. The fitted Gaussian curve (mean = 0.760 points/mm^2^, SD = 0.298 points/mm^2^) describes this central cluster but fails to capture the distinct tail extending toward lower densities (0.15–0.45 points/mm^2^). This separation supports the use of a density threshold to isolate regions affected by genuine geometric occlusion.

**Figure 15:**
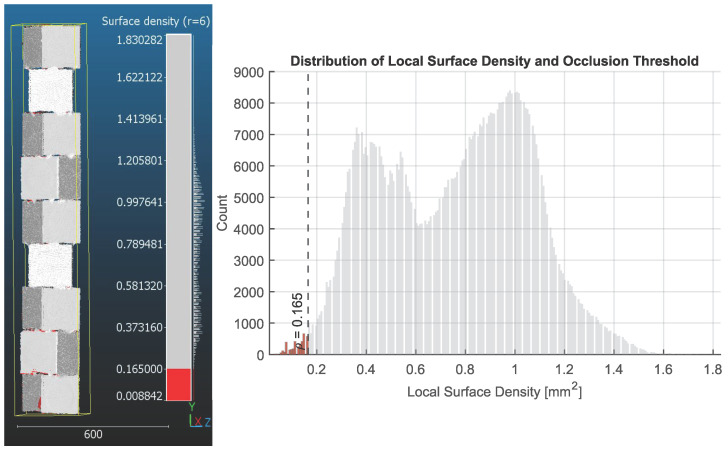
The segmented low-density regions based on the 0.165 mm^2^ threshold. Points below this threshold are shown in red and cluster along corners, recessed joints, and stepped surfaces where scanner visibility is reduced (**left**). The histogram of low-density values indicates how the selected threshold relates to the overall distribution and highlights the separation between well-observed and partially occluded regions (**right**).

Segmentation of points falling below the 10th percentile threshold (0.165 points/mm^2^) identifies approximately 4.2% of the total surface area as sparsely sampled. As shown in Figure 15, these points (highlighted in red) are spatially concentrated along sharp edges, corners, and the undersides of stepped layers.

These results reflect the intrinsic line-of-sight constraints of the single-sensor acquisition path. Although the scanner performs a complete 360° rotation, inward-facing joints and surfaces tilted away from the sensor remain geometrically concealed across multiple viewpoints. Vertical merging cannot resolve these gaps, as the visibility limitations persist across all elevation steps. Consequently, the low-density clusters represent true visibility limits rather than inconsistencies in motion control. The recessed undercuts and sharp $90^{{}^{\circ}}$ overhangs of the modular benchmark represent a ‘worst-case’ visibility scenario. In contrast, the organic topology of a standing human subject is predominantly convex and continuous, minimising the occurrence of such hard occlusion zones. Consequently, the coverage limitations observed here are likely exaggerated compared to the system’s performance on biological forms. The stability of the high-density peak (0.7–$1.2\;{\mathrm{points}/\mathrm{mm}}^{2}$) confirms that for all accessible surfaces, the system maintains uniform sampling coverage.

### 3.2. Geometric Accuracy and Volumetric Error

The quantitative comparison against the CAD reference provides a measure of the system’s volumetric fidelity. However, scalar summary statistics alone do not reveal the spatial structure of accumulated drift during sequential registration. In particular, unconstrained ICP is known to suffer from telescoping effects when overlapping frames are aligned over extended trajectories with repetitive geometry.

**Figure 16:**
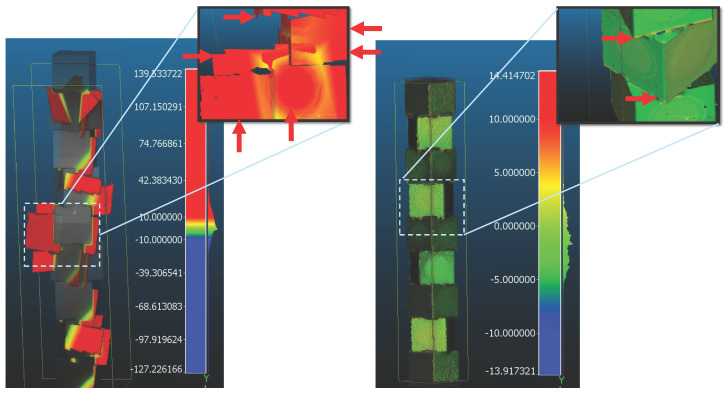
Cloud-to-mesh deviation comparison between raw ICP (**left**) and sensor-assisted ICP (**right**) reconstructions relative to the CAD benchmark. Signed C2M deviations are shown using an identical colour scale (±10 mm) for both reconstructions. Red arrows indicate representative regions where the ICP reconstruction exceeds 5 mm deviation, highlighting telescoping drift accumulation. Corresponding close-up views are shown for both reconstructions to illustrate the suppression of drift at stepped joints and top-layer cube edges under sensor assistance.

To explicitly visualise this behaviour, a focused C2M deviation comparison was performed between raw ICP and sensor-assisted ICP reconstructions relative to the CAD benchmark as shown in Figure 16. Both reconstructions were rigidly aligned to the CAD using ICP, and signed C2M deviation maps were computed using identical parameters. A fixed signed color scale was applied to both reconstructions to preserve spatial error structure while avoiding saturation effects associated with narrow display ranges.

A deviation magnitude of 5 mm was adopted as a pragmatic threshold for identifying structurally meaningful telescoping drift, exceeding both scanner noise and local surface roughness. Representative regions in the raw ICP reconstruction exceeding this threshold are contrasted against the corresponding regions in the sensor-assisted reconstruction, where substantially reduced deviation is observed. These visualisations directly illustrate the anti-telescoping effect achieved by the proposed sensor-assisted pipeline.

**Figure 17:**
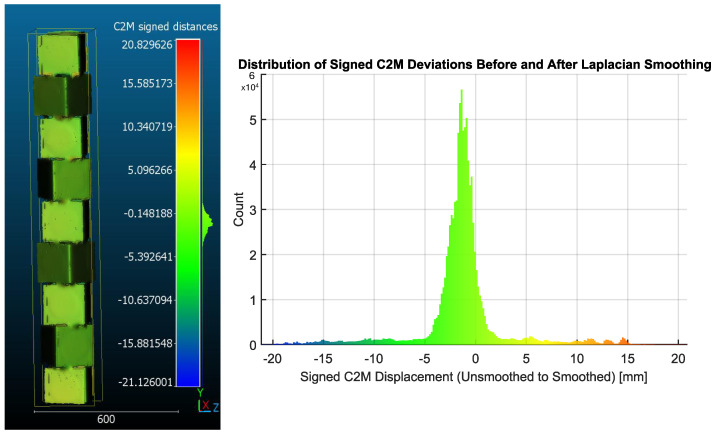
Quantification of Laplacian smoothing-induced shrinkage. Cloud-to-mesh deviation map (**left**) between the reconstructed surface before and after Laplacian smoothing, showing predominantly inward displacement. Histogram of the signed C2M deviations (**right**), yielding a mean of −0.72 mm and a standard deviation of 0.81 mm. This indicates systematic surface contraction induced by smoothing, accounting for approximately 47% of the observed −1.54 mm global bias relative to the CAD benchmark.

To isolate post-processing bias, a C2M comparison, as shown in Figure 17, was performed between the reconstructed surface before and after Laplacian smoothing. The resulting deviation field exhibited a predominantly inward displacement, with a mean signed deviation of −0.72 mm and a standard deviation of 0.81 mm, indicating systematic surface contraction induced by smoothing.

This confirms that a substantial fraction of the observed −1.54 mm global bias arises from post-processing rather than registration error. Specifically, Laplacian smoothing alone accounts for approximately 47% of the reported inward bias.

**Figure 18:**
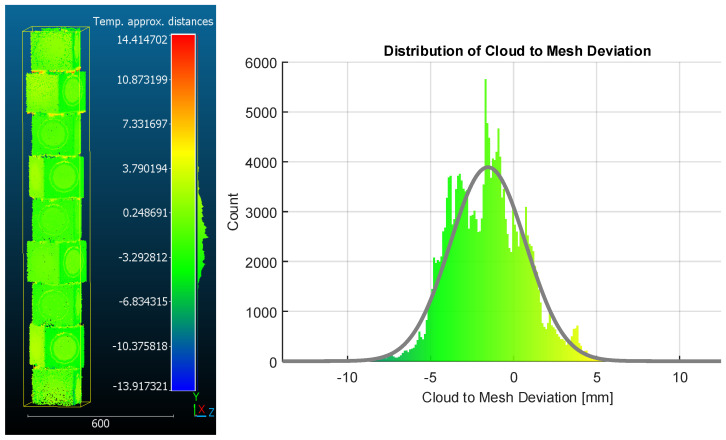
Cloud-to-mesh deviation analysis. The signed deviation map highlights local agreement between the scan and the CAD model, with most surfaces showing small errors and larger deviations restricted to edges and occluded regions (**left**). The corresponding histogram with Gaussian fit (mean = −1.54 mm, σ= 2.27 mm) shows a narrow central distribution with a long positive tail arising from under-sampled features (**right**).

Figure 18 illustrates the spatial distribution of signed deviations, where the majority of the surface exhibits tight agreement with the reference geometry.

The distribution of C2M distances shows a clear separation between typical surface noise and outliers driven by occlusion. The Gaussian fit yields a mean signed deviation of −1.54 mm and a standard deviation of 2.27 mm. The presence of a negative bias indicates a slight volumetric contraction of the reconstructed model relative to the reference geometry.

**Figure 19:**
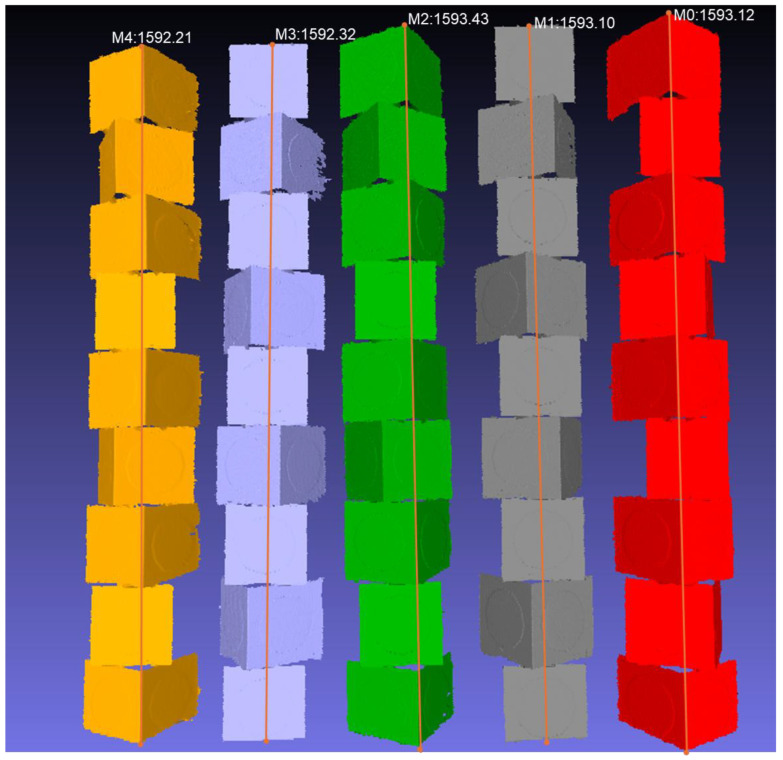
End-to-end reconstruction repeatability across five independent acquisitions (M0–M4) under identical scanning conditions. Each column shows the reconstructed benchmark from a separate run. The orange line indicates the extracted reconstructed vertical extent of each run. The measured reconstructed heights span 1592.21–1593.43 mm (peak-to-peak variation 1.22 mm; σ = 0.49 mm), corresponding to a relative variation of approximately 0.031% (1σ) over a ∼1.6 m object.

To assess whether the reported volumetric behaviour reflects stable system performance rather than a single-run outcome, five independent acquisitions, as shown in Figure 19, of the benchmark were processed using the same reconstruction pipeline and identical registration parameters. For each run, the reconstructed complete vertical extent was extracted using consistent geometric reference landmarks.

It is important to note that this height metric represents the end-to-end output of the complete acquisition and reconstruction process, rather than a direct physical measurement of the benchmark. The values therefore incorporate the combined effects of benchmark assembly tolerance, sensor noise, motion control uncertainty, ICP convergence variability, and post-processing bias. From a repeatability perspective, however, this end-to-end measure is the quantity of interest. Height was extracted as the distance between the extremal points of the reconstructed model along the global vertical axis after applying the same outlier filtering and alignment procedure for all runs.

Across the five runs, the reconstructed height varied from 1592.21 mm to 1593.43 mm, corresponding to a peak-to-peak variation of 1.22 mm and a standard deviation of 0.49 mm over a 1593 mm object. This corresponds to a relative variation of approximately 0.031% (1σ), demonstrating that the reconstruction behaviour is stable across independent acquisitions. The observed spread is consistent with independently quantified systematic effects (such as modular cube manufacturing variation and smoothing-induced shrinkage), indicating that the reported volumetric error is not driven by random run-to-run instability.

While the Artec Eva Lite possesses a specified point accuracy of 0.1 mm, this specification applies to local geometry within a single frame. For large-scale reconstruction, volumetric error accumulation becomes the dominant factor. Over the 1.6 m vertical span of the target object, the mean deviation of −1.54 mm corresponds to a relative dimensional error of approximately 0.096%. This negative bias (<1 mm per meter) is attributed to three concurrent factors (noting that physical manufacturing bias was negligible):

- Volumetric Drift: Without external photogrammetry markers (global registration), sequential ICP alignment over long distances typically accumulates slight translational errors, often manifesting as linear compression in the dominant axis of travel.
- Smoothing Artefacts: The Laplacian smoothing applied during post-processing inherently causes mesh shrinkage, particularly on convex features like the cube corners.
- Optical Edge Erosion: The structured-light sensor samples sharp depth discontinuities using a finite projected pattern and camera pixel grid, which inherently smooths step edges into rounded fillets at sub-pixel scale. This effect has been reported for structured-light systems and leads to a systematic loss of apparent volume at sharp corners, contributing to the residual inward bias not accounted for by smoothing alone [30].

Global statistics, which include outliers, show a maximum deviation of 12.46 mm. However, these larger values are spatially localised to sharp edges and recessed joints where grazing angle acquisition leads to higher registration uncertainty. Importantly, the minimum deviation approaches the limits of resolution (<0.01 mm), confirming that substantial portions of the planar faces align closely with the CAD reference.

Considering the absence of external tracking hardware, a volumetric error below 0.1% demonstrates that the mechanically guided trajectory successfully mitigated the non-linear bending and warping artefacts typically associated with handheld scanning of tall objects. The analysis confirms that the system achieves high volumetric fidelity suitable for anthropometric measurement, with accuracy primarily governed by line-of-sight constraints rather than algorithmic instability.

While this study utilised rigid geometric benchmarks, the proposed pipeline is inherently capable of reconstructing organic forms. The primary challenge in scanning human anatomy is the lack of distinct geometric features (e.g., cylindrical limbs), which the sensor-assisted initialisation strategy was explicitly designed to resolve. Furthermore, the geometric contraction observed in this study was largely driven by the effect of Laplacian smoothing on sharp synthetic corners. For organic subjects, which possess continuous surface curvature, this smoothing artefact is expected to be significantly reduced. Regarding material properties, the standard PLA used for the benchmarks presents a semi-gloss surface that is optically conservative compared to the diffuse reflectance of dry human skin, which is generally ideal for structured-light acquisition. Most importantly, the use of a static rigid benchmark was a metrological necessity; evaluating system-level precision (sub-millimetre) requires a target free from the postural sway and respiratory motion inherent in live subjects.

## 4. Conclusions and Future Works

This study presented the development and evaluation of a low-cost, automated 3D scanning platform tailored for human-scale reconstruction. By integrating a single commercial sensor with a mechanically constrained linear–rotational trajectory, the system addresses the critical challenges of geometric ambiguity and operator variability inherent in handheld scanning. The implementation of a sensor-assisted initialisation strategy, coupling hardware encoders with specific registration constraints, was shown to effectively eliminate the “telescoping” alignment failures typically observed when scanning repetitive vertical structures.

The experimental evaluation using modular anthropometric benchmarks demonstrated that the system achieves industrial-grade stability. The acquisition path provided uniform sampling density ($0.760\;{\mathrm{points}/\mathrm{mm}}^{2}$) across all front-facing planar surfaces, with under-sampled regions confined to predictable line-of-sight occlusions such as recessed joints. In terms of volumetric fidelity, the system achieved a mean geometric deviation of −1.54 mm over a ∼1.6 m vertical span, corresponding to a relative volumetric error of approximately 0.096%. While this negative bias is largely attributed to grazing angle acquisition and post-processing smoothing shrinkage, the analysis also identified discrepancies arising from systematic optical artefacts. Specifically, the finite optical resolution of the sensor inherently rounds the sharp orthogonal edges of the benchmark, resulting in a systematic loss of edge volume relative to the ideal CAD geometry. These edge-specific artefacts contributed to deviations from the ideal CAD reference, highlighting the sensitivity of the system to even minor surface irregularities. Collectively, these results confirm that a mechanically guided single-sensor system can mitigate the non-linear bending and drift artefacts of handheld scanning, producing a stable reconstruction suitable for anthropometric measurement.

Future work will focus on enhancing both the physical hardware and the algorithmic pipeline. To address the identified assembly tolerances, future iterations of the benchmark objects will utilise higher-precision manufacturing methods to ensure perfectly flush interlocking interfaces. From an algorithmic perspective, the research will pivot towards implementing a robust geometric calibration framework. While the current study relies on pairwise registration to stitch consecutive frames, future research will utilise ICP to explicitly calculate the fixed alignment parameters between the scanner’s optical centre and the motion axes. Once this geometric relationship is established, the system can rely primarily on encoder feedback to position the point clouds in 3D space, effectively eliminating the need for computationally intensive alignment steps during the acquisition phase. Instead, ICP will be applied only as a final refinement step. Subsequent studies will quantify the accuracy of this calibrated workflow and compare its performance against the baseline established in this work. Finally, while this work validated the platform using rigid benchmarks, a follow-up study will aim to extend this evaluation to human subjects. This future work will specifically address the optimisation of acquisition parameters to minimise scan time while maximising surface fidelity on organic, non-rigid topologies.

## Data Availability

The data that support the findings of this study will be made available in a public repository following an embargo period due to ongoing research and potential commercialisation of the methods and dataset. In the interim, minimal datasets can be made available for verification purposes upon reasonable request to the corresponding authors.
